# Acidic ascites inhibits ovarian cancer cell proliferation and correlates with the metabolomic, lipidomic and inflammatory phenotype of human patients

**DOI:** 10.1186/s12967-022-03763-3

**Published:** 2022-12-12

**Authors:** Qianlu Yang, Gyuntae Bae, Giorgi Nadiradze, Arianna Castagna, Georgy Berezhnoy, Laimdota Zizmare, Aditi Kulkarni, Yogesh Singh, Frank J. Weinreich, Stefan Kommoss, Marc A. Reymond, Christoph Trautwein

**Affiliations:** 1 National Center for Pleura and Peritoneum, NCT South-West Germany, Tübingen, Germany; 2 Present Address: Department of Preclinical Imaging and Radiopharmacy, Werner Siemens Imaging Center, University Hospital Tübingen, Tübingen, Germany; 3 Department of General and Transplant Surgery, University Hospital Tübingen, Tübingen, Germany; 4 Institute of Medical Genetics and Applied Genomics, University Hospital Tübingen, Tübingen, Germany; 5 Research Institute of Women’s Health, Women’s Hospital, University Hospital Tübingen, Tübingen, Germany

**Keywords:** Peritoneal fluid, In vitro, In vivo, pH, Cell culture, Metabolic profile, Cytokine

## Abstract

**Background:**

The poor prognosis of ovarian cancer patients is strongly related to peritoneal metastasis with the production of malignant ascites. However, it remains largely unclear how ascites in the peritoneal cavity influences tumor metabolism and recurrence. This study is an explorative approach aimed at for a deeper molecular and physical–chemical characterization of malignant ascites and to investigate their effect on in vitro ovarian cancer cell proliferation.

**Methods:**

This study included 10 malignant ascites specimens from patients undergoing ovarian cancer resection. Ascites samples were deeply phenotyped by ^1^H-NMR based metabolomics, blood-gas analyzer based gas flow analysis and flow cytomertry based a 13-plex cytokine panel. Characteristics of tumor cells were investigated in a 3D spheroid model by SEM and metabolic activity, adhesion, anti-apoptosis, migratory ability evaluated by MTT assay, adhesion assay, flowcytometry and scratch assay. The effect of different pH values was assessed by adding 10% malignant ascites to the test samples.

**Results:**

The overall extracellular (peritoneal) environment was alkaline, with pH of ascites at stage II-III = 7.51 ± 0.16, and stage IV = 7.78 ± 0.16. Ovarian cancer spheroids grew rapidly in a slightly alkaline environment. Decreasing pH of the cell culture medium suppressed tumor features, metabolic activity, adhesion, anti-apoptosis, and migratory ability. However, 10% ascites could prevent tumor cells from being affected by acidic pH. Metabolomics analysis identified stage IV patients had significantly higher concentrations of alanine, isoleucine, phenylalanine, and glutamine than stage II-III patients, while stage II-III patients had significantly higher concentrations of 3-hydroxybutyrate. pH was positively correlated with acetate, and acetate positively correlated with lipid compounds. IL-8 was positively correlated with lipid metabolites and acetate. Glutathione and carnitine were negatively correlated with cytokines IL-6 and chemokines (IL-8 & MCP-1).

**Conclusion:**

Alkaline malignant ascites facilitated ovarian cancer progression. Additionally, deep ascites phenotyping by metabolomics and cytokine investigations allows for a refined stratification of ovarian cancer patients. These findings contribute to the understanding of ascites pathology in ovarian cancer.

**Supplementary Information:**

The online version contains supplementary material available at 10.1186/s12967-022-03763-3.

## Introduction

Ovarian cancer (OC) is the most common form of cancer among women worldwide with thousands of deaths every year [1]. The poor prognosis of patients relates to the rapid progression of peritoneal metastasis [2]. Peritoneal metastasis develops following detachment of tumor cells from their primary tumor, gain of motility, apoptosis evasion, adherence to the peritoneal surface, invasion into the peritoneum, proliferation and accelerated growth [3]. The resting metabolism increases along with peritoneal metastasis progression, leading to cachexia, anorexia, and finally, the death of patients [4, 5]. The most common primary cancer that is associated with the production of ascites is ovarian cancer, accounting for 38% of malignant ascites occurring in females [6]. In advanced stages of OC, such as the International Federation of Gynecology and Obstetrics (FIGO) stages III and IV, metastases into the abdominal cavity can be often observed alongside an ongoing inflammatory process which contributes to the production of ascites [7]. Ascites acts as a fluid microenvironment that influences carcinogenesis. Various factors of the microenvironment, such as pH or oxygen levels, as well as concentrations of metabolites excreted by surrounding tissues, can cause alterations and mutations in cancer cells, leading to increased tumor heterogeneity [8]. For example, disturbed pH delays cell entry into G2/M by limiting the activity of cell cyclin-dependent kinase 1 (CDK1)–cyclin B1; it also inhibits mitotic arrest triggered by activated DNA damage checkpoints, thereby promoting unrestricted proliferation as well as genetic instability [9]. Moreover, altered angiogenesis and lymphangiogenesis in tumor cells, tumor metabolism, growth and treatment resistance can be mediated by the transcription factor hypoxia-inducible factor 1⍺ (HIF-1⍺), which is activated by hypoxia [10]. Of note, ascites may contain markers of the neoplastic process way earlier than peripheral blood plasma and thereof offers a high potential for diagnostic purposes [11].

The mechanism underlying peritoneal metastases and carcinomatosis is complex. Cancer cells which detach from the primary tumor into the peritoneal cavity lose contact with the extracellular matrix (ECM) or neighboring cells [12]. In order to survive in the hostile peritoneal environment, they often develop resistance to anoikis ("anoikis" means "homeless" in ancient Greek) by starting a cascade of metabolic adaptations [13]. These metabolic alterations, after detachment from the ECM, include defective glucose uptake, diminished pentose phosphate pathway (PPP) flux, reduced cellular adenosine triphosphate (ATP) levels, and an increase in mitochondrial-generated reactive oxygen species (ROS) [14]. Specifically, solitary tumor cells switch their hexokinase metabolism from mitochondrial oxidation to aerobic cytosolic glycolysis, well-known as Warburg effect. First described by Otto Warburg [15], this effect refers to the observation that cancer cells prefer cytosolic glycolysis even in aerobic conditions because it is 10–100 times more rapid to produce ATP. Later, in metastatic progression, detached cancer cells recruit normal cells from the host and form "metastatic units" with cancer-associated fibroblasts (CAFs). The association with CAFs supports energy metabolism of cancer cells by supplying lactate and amino acids [16].

To date it remains largely unclear how the production of ascites in the peritoneal cavity influences tumor metabolism and recurrence. Thereof, we used an explorative approach of pH dependencies and various state-of the art techniques such as flow cytometry-based cytokine assay and nuclear magnetic resonance (NMR)-based metabolomics analysis to deeper characterize ascites and determine the effect of ascites on ovarian cancer.

## Methods

This study included 10 malignant ascites specimens from patients undergoing ovarian cancer resection. By adjusting the pH of the cell culture medium, 3D spheroid cells and ovarian tumor cells were cultured, morphological changes were examined by scanning electron microscopy (SEM), and 3-(4,5-dimethylthiazol-2-yl)-2,5- diphenyltetrazolium bromide (MTT) assay, adhesion assay, flow cytometry, and scratch assay were used to evaluate metabolic activity, adhesion, anti-apoptosis, and migration abilities of ovarian tumors at various pH. The effect of ascites on tumor cells at different pH was assessed by adding 10% of malignant ascites to the test samples. Blood-gas analyzer for airflow analysis of ascites, ^1^H-NMR for metabolomic analysis, and flow cytometry for cytokine analysis were used to combine the results for in-depth phenotypic analysis of ascites.

### Ethical background

This study was approved by the Ethics Committee, Faculty of Medicine, University of Tübingen, Germany (Ref. Nr. 696/2016BO2 and 117/2020BO1). All patients gave the written informed consent. Sampling did not influence patient treatment. All data were anonymized according to European Data Regulation Regulations (EDPR) and German law.

### Collection and storage of patient specimens

Ascites samples were collected from patients undergoing surgery for ovarian cancer at the department of General and Transplant Surgery and at the Women's Hospital, University Hospital Tübingen, Germany. Ascites probes were sampled in the operating room, stored in tubes, and transferred to the laboratory using an ice box. The collected samples were divided into 1 mL tubes and frozen directly in a − 80 °C refrigerator for physical–chemical parameter measurements. The remaining ascites was first centrifuged at 4 °C for 30 min at 1200 rpm and stored in 2 mL tubes at − 80 °C until analysis. Patient information was collected, including standard demographics, cancer histology, and the extent of peritoneal disease. Samples and data were anonymized.

### Cell culture and treatment

Human ovarian cancer cells (OAW42, German Cancer Research Center (DKFZ), Heidelberg, Germany) were cultured in DMEM (Thermo Fisher, New York, USA). Monolayered cells were cultured in 175 cm^2^ flasks (Falcon, Corning, New York, USA) supplemented with 10% fetal bovine serum and penicillin G 100 U/mL, streptomycin 100 μg/mL in a humidified atmosphere of 5% CO_2_, 95% air at 37 °C. For experimental purposes, cells were cultured in 24-well plates (1.5 mL of cell culture medium or 10% ascites/well) under different pH conditions (pH 6.0, 6.5, 7.0, 7.5) (using phosphate buffered saline (PBS) and phosphoric acid or sodium hydroxide (NaOH)). pH was monitored using a pH meter (SevenCompact™ Duo pH/Conductivity S213, Mettler-Toledo GmbH, Greifensee, Switzerland).

### 3D spheroid cell model

We first established 3D spheroid-matrigel-based models, in which cell aggregates are grown in suspension. OAW42 cell suspensions were mixed with Matrigel (A1413201, Thermo Fisher, USA), seeded into 96 U-Form plates, and centrifuged at 2,000 rpm for 10 min at 4 °C. Then, 3D spheroids were incubated in a standard cell culture medium for seven days. After 7 days, 3D spheroids were transported to glass plates in 24 well plates, and the cell culture medium was titrated to different pH conditions (6.0, 6.5, 7.0, 7.5) for seven days. The pH was titrated with phosphoric acid or NaOH to reach pre-specified extracellular pH conditions (6.0, 6.5, 7.0, 7.5). The cell culture medium was changed every two days. After seven days of culture in different pH ranges, samples were fixed in 4.5% formaldehyde/2.5% glutaraldehyde in 0.1 M PBS at 4 °C overnight, washed with PBS, post-fixed with 1% osmium tetroxide in PBS, and dehydrated by 30%, 50%, 70%, 95%, and 100% ethanol (EtOH). Then, samples were placed in a critical point dryer (EM CPD300, Leica Microsystems, Wetzlar, Germany) and coated with platinum in a sputter (EM ACE200, Leica Microsystems, Wetzlar, Germany). The dried and coated samples were then observed by SEM (Phenom G2pro and Software Phenom ProSuite, Phenom-World BV, Eindhoven, The Netherlands).

### Cell metabolic activity assay

After seeding OAW42 cells, the pH of the cell culture medium was titrated daily to remain at a constant pH value which was measured by a conventional pH meter (SevenCompact™ Duo pH/Conductivity S213, Mettler-Toledo GmbH, Greifensee, Switzerland). The metabolic activity of OAW42 cells was determined by a MTT Assay (R&D Systems, 4890-050-k, Germany). Viable cancer cells contain NAD(P)H-dependent oxidoreductase enzymes which reduce a tetrazolium salt MTT to formazan. The MTT assay is a colorimetric assay based on the yellow MTT changing to purple formazan crystals in metabolically active cells. After seeding every cell line to three 96-wells plates overnight. One plate was incubated for four hours at 37 °C with 20 μL MTT reagent (R&D Systems, 4890-25-01, Germany) until the purple dye was visible. 100 µL of detergent reagent (R&D Systems, 4890-25-02, Germany) was added, and the cells were kept at RT overnight. The following day, the absorbance in each well was measured at 560 nm using the NanoQuant Infinite M200 Pro microplate reader (Tecan Austria GmbH, Grödig, Austria). Half of the remaining two 96-well plates were supplemented with 10% human ascites. The MTT assay was repeated after 24 h and 48 h.

### Adhesion assay

The influence of pH on the adhesion of OAW42 cells was determined in vitro by a colorimetric cell adhesion assay (The CytoSelect 48-Well Cell Adhesion Assay Kit, CBA-070, USA). The absorbance was determined with the NanoQuant Infinite M200 Pro microplate reader (Tecan Austria GmbH, Grödig, Austria). After titrating the pH of the cell culture medium and supplementing 50% of the samples with 10% human ascites, OAW42 cells were grown in the incubator for 12, respectively, 24 h. Cells were suspended in serum-free media, and the suspension was pipetted into 40 ECM protein-coated (Fibronectin, Collagen I, Collagen IV, Laminin I, Fibrinogen) wells and 8 Bovine serum albumin (BSA)-coated wells and then incubated in a cell culture incubator. After PBS-washing, cells were stained by Cell Stain Solution (The CytoSelect 48-Well Cell Adhesion Assay Kit, CBA-070, USA). Then, cells were washed with deionized water, the supernatant extracted, and cells were transferred to a 96-well plate. Optical density was then measured by NanoQuant Infinite M200 Pro microplate reader (Tecan Austria GmbH, Grödig, Austria) at a wavelength of 560 nm.

### Flow cytometry assay

The effect of increasing or reducing the extracellular pH in ascites on cell resistance to apoptosis of OAW42 cells was measured in vitro by flow cytometry (FACS). Apoptosis was measured with the Annexin V- Fluorescein (FITC) Apoptosis detection kit (Thermo Fisher, BMS500FI, USA). OAW42 cells were kept in a cell culture medium at different pH for 12, respectively, 24 h. 50% of the wells were supplemented with 10% human ascites. Cold PBS and binding buffer washed resuspend cells, then mixed with Annexin V-FITC and PI at RT. According to the protocol of the manufacturer, cells were stained with propidium iodide (PI)/annexin V-FITC (annexin V-fluorescein isothiocyanate). Stained cells were acquired on flow cytometry (FACS Canto II, Becton Dickinson GmbH, Heidelberg, Germany).

### Wound scratch assay

The migratory ability of cancer cells at different extracellular pH was measured using a scratch wound assay. OAW42 cells were seeded in a 24-well plate overnight. A standardized wound was scratched with a 10 ul pipette. Cells were washed twice with PBS, half of the wells were supplemented with 10% human ascites, and the cell culture medium was titrated to different pH (6.0 6.5 7.0 7.5). The plates were placed into an incubator at 37 °C, and cell migration was monitored in real-time by micro-cinematography (zen Cell-Owl, Bremen, Germany). The extent of cell migration at 6, 24 and 36 h was expressed as the wound width of the scratch relative to the initial scratch, expressed as %.

### Gene expression omnibus (GEO) databases

The mRNA expression comparison with primary and ascites cells was done by downloading respective data sets from the GSE73064 database [17] and processing them by standard methods. To characterize the effect of ascites on ovarian cancer cells, we performed a differential analysis (Log_2_|FC|> 1, *p* < 0.05) by comparing ascites cancer cells to primary cancer cells by R language using the "limma" package. In the gene set enrichment analysis (GSEA) [18], the statistical significance was defined as *p* < 0.05, and the overrepresentation of indicated hallmark gene sets in the ranked gene lists presented by the normalized enrichment score (NES). Gene ontology (GO) analyses were conducted for the selected common differentially expressed genes (DEGs) using the R language, *p* < 0.05.

### Physical–chemical characterization of ascites

150 µl of each ascites sample were taken for a measurement of an industry-standard blood gas analyzer (GEM PREMIER4000, Werfen, Germany). Outcome parameters were pH, pCO_2_, glucose, lactate, and electrolytes (Na^+^, K^+^, Ca^2+^, Cl^−^).

### ^1^H-NMR spectroscopy equipment and spectra acquisition

^1^H-NMR spectroscopy (Bruker Avance III HD) was operated at 600 MHz (14.1 Tesla) with a 3.0 mm probe at 298 K using Carr-Purcell-Meiboom-Gill pulse programs (CPMG). CPMG spectra were pre-processed by Bruker TopSpin 3.6.1 and quantified using Chenomx NMR suite 9.02 software.

### Metabolite extraction of ascites for metabolomics analysis

All the ascites of OCs were thawed, and 2 mL of each ascites was pipetted to the Eppendorf tube for centrifugation at 30,000G for 10 min. 500 μL of each supernatant were transferred to a Covaris tube (Covaris, Woburn, USA) and placed in SpeedVac (Thermo Fisher, SPD300DDAA-230, USA) to evaporate unwanted solvents for 3 h. 300 μL of methanol and tert-butyl methyl ether (MTBE) were added to the Covaris tube using filter-containing pipette tips for total lipid extraction and vortexed into a homogeneous solution. A total of 5 min ultrasound extraction was used for each sample in the Covaris Ultrasonicator E220 Evolution instrument (Covaris, Woburn, USA). After ultrasonication, 250 μL of molecular biology water (ultra-pure grade) were added and tubes were centrifuged at 12,000G for 10 min to obtain polar and non-polar (lipid) phases. Lipid and polar phase were separated to glass vials and Polytetrafluorethylene (PTFE) tubes, respectively.

### Metabolomics analysis of polar metabolites by ^1^H-NMR spectrometry

210 μL of deuterated 1 mM 3-(trimethylsilyl)propionic-2,2,3,3 acid sodium salt D4 (TSP-D4) buffer (K_2_HPO_4_ in D_2_O + 10 mM NaN_3_), adjusted to pH 7.44, was pipetted to each polar sample dried pellet in Eppendorf tube. The Eppendorf tubes were vortexed until the samples were dissolved and then bathed under ultrasound for seconds. Next, the PTFE tube was centrifuged at 30,000G for 30 min. The supernatants were pipetted to a 3 mm NMR spectrometer compatible tube (Bruker Biospin, Reinstatten, Germany) using two pipetting steps with 95 μL each. All the NMR tubes were then fixed with a cap sealing ball and wiped with fusel-free tissue right before placing them inside the NMR spectrometer autosampler unit. The polar samples spectra were recorded using Nuclear Overhauser Spectroscopy (NOESY 1D) and CPMG experiments; the duration of 7 min and 2 h, respectively. The CPMG spectra were chosen for analysis due to the better signal-to-noise ratio (SNR) and lipid signal suspression compared to 1D NOESY spectra.

### Lipid metabolite sample preparation for metabolomics analysis

500 μL of each lipid aliquot in MTBE solvent was transferred to a glass high performance liquid chromatography (HPLC) vial and evaporated to dryness in a vacuum dryer 200 μL of deuterated chloroform solution with an internal 1 mM tetramethylsilane (TMS) standard was added to the dried lipid pellet in a glass vial, and thoroughly mixed vortexed. 200 μL of each sample was pipetted into a 3 mm NMR tube using a round solvent-safe tip; 100 μL of each lipid sample was pipetted twice. All the NMR tubes were then fixed with a cap sealing ball and wiped with fusel-free tissue right before placing them inside the NMR autosampler. Lipid samples were analyzed by ^1^H NMR using a 1'hour lasting simple proton experiment (zg30) and 1 h lasting J-coupling resolved spectroscopy (JRES) experiment. Proton experiments were chosen for spectral assignments of lipid metabolites.

### Quantification of cytokines present in malignant ascites

To measure the cytokines levels, 25 μL of ascites of OCs was mixed with 25 μL of assay buffer. Then, 25 μL of 13-plex-beads were pipetted to a 96-well microplate (LEGENDplex™ Human Inflammation Panel 1 (13-plex) #740,809, BioLegend, USA). This assay can quantify a total of 13 cytokines/chemokines (the minimum detectable concentration (MDC) in brackets: IL-1β (1.5 + 0.6 pg/ml), IFN-α2 (2.1 + 0.2 pg/ml), IFN-γ (1.3 + 1.0 pg/ml), TNF-α(0.9 + 0.8 pg/ml), MCP-1 (1.1 + 1.2 pg/ml), IL6 (1.5 + 0.7 pg/ml), IL-8 (2.0 + 0.5 pg/ml), IL-10 (2.0 + 0.5 pg/ml), IL-12p70 (2.0 + 0.2 pg/ml), IL17A (0.5 + 0.pg/ml), IL-18 (2.0 + 0.5 pg/ml), IL-23 (1.8 + 0.1 pg/ml), IL-33 (4.4 + 1.5 pg/ml)). The microplate was then incubated and shaken for 2 h at room temperature in which the analytes (cytokines) bound to an antibody-conjugated capture bead. After washing, biotinylated detection antibodies (25 μL) were added and bound to the analytes. Streptavidin–phycoerythrin (25 μL) was subsequently added that bound to the antibodies and provided a fluorescent signal with intensities in proportion to the amount of the bound analyte. After 1 h incubation, beads were washed and flow cytometer was used to quantified the fluorescent signal, and concentrations of the analytes were determined based on a known standard curve using LEGENDplex™ data analysis software (BioLegend, USA).

### Chemometrics

All the data were normalized to a pooled sample from group probabilistic quotient normalization (PQN), and log-transformation was applied. Univariate, multivariate, and correlation analysis was performed using the MetaboAnalyst 5.0 toolbox (https://www.metaboanalyst.ca), where due to the low number of total samples the comparison was made based on combining clinical stage II-III versus stage IV.

### Univariate analysis

The volcano plot was used to analyze the polar and lipid metabolites present in the OC with II-IV. A value of *p* < 0.05 and fold change (FC) cut-off > 1.2 was considered significant.

### Multivariate analysis

Principle component analysis (PCA) and orthogonal projections to latent structures discriminant analysis (OPLS-DA) score plots were used to see how each OC stage samples clustered to another. PCA and OPLS-DA loading plots and PCA bi-plot were further analysed for specific metabolite involvement in OC progression. The correlation analysis was carried out to observe correlations between certain lipid metabolite species, polar metabolite, pCO_2_, pH, and cytokines. The selected cytokines and polar metabolites were IL-8, glutathione, acetate, 3-hydroxybutyrate, glycerol, and lactate, respectively.

### Statistics

This was an exploratory study without prior sample size calculation. Data were first visualized with pair plots (Seaborne, Anaconda, Berlin). Descriptive statistics and correlations were calculated with R, GSEA, Prism GraphPad, and SPSS software. Comparative statistics were performed using t-test, one-way analysis of variance (ANOVA) and two-way ANOVA for normally distributed data, and non-parametric tests for skewed data. Graphical abstract created with BioRender.com.

## Results

### Patients clinical information

Ascites samples were collected from ovarian cancer patients who required open surgery to remove gynecologic malignancies. Table 1 illustrates the clinic-pathological characteristics of the patients. Prior to ascites collection, none of the patients received any treatment. The patient with clear cell carcinoma did not have endometriosis.

**Table 1:** Clinic-pathological characteristics of the explorative cohort

Number of patients	10
Age (mean ± SD)	65.30 ± 9.67
Gender	Female
Cancer type	Ovarian cancer
BMI (Kg/m2) (mean ± SD)	25.16 ± 8.86
ECOG	
0	6 (60%)
1	2 (20%)
2	1 (10%)
3	1 (10%)
Histology	
High-grade serous carcinoma	8 (80%)
Low-grade serous carcinoma	1 (10%)
Clear cell carcinoma	1 (10%)
Federation of gynecology and obstetrics stage (FIGO)	
Stage II	1 (10%)
Stage III	6 (60%)
Stage IV	3 (30%)
T Stage	
T2	1 (10%)
T3	7 (70%)
Tx	2 (20%)
N Stage	
N0	3 (30%)
N1	5 (50%)
Nx	2 (20%)
M Stage	
M0	4 (40%)
M1	2 (20%)
Mx	4 (40%)
Primary surgery	
Stage II	1 (100%)
Stage III	5 (83%)
Stage IV	2 (66%)
Residual disease	
Stage II	R0: 1 (100%)
Stage III	R0: 2 (33%), R1: 3 (60%), R2: 1 (17%)
Stage IV	R2: 3 (100%)

Residual disease after surgery was reported as R0, R1, and R2. R0 was defined as no macroscopic residual disease. R1 and R2 were defined as macroscopic residual disease with a maximal diameter of < 1 cm and > 1 cm, respectively.

### The acidic extracellular environment inhibits cancer cell growth, but malignant ascites is resistant to this inhibition effect

**Fig. 1:**
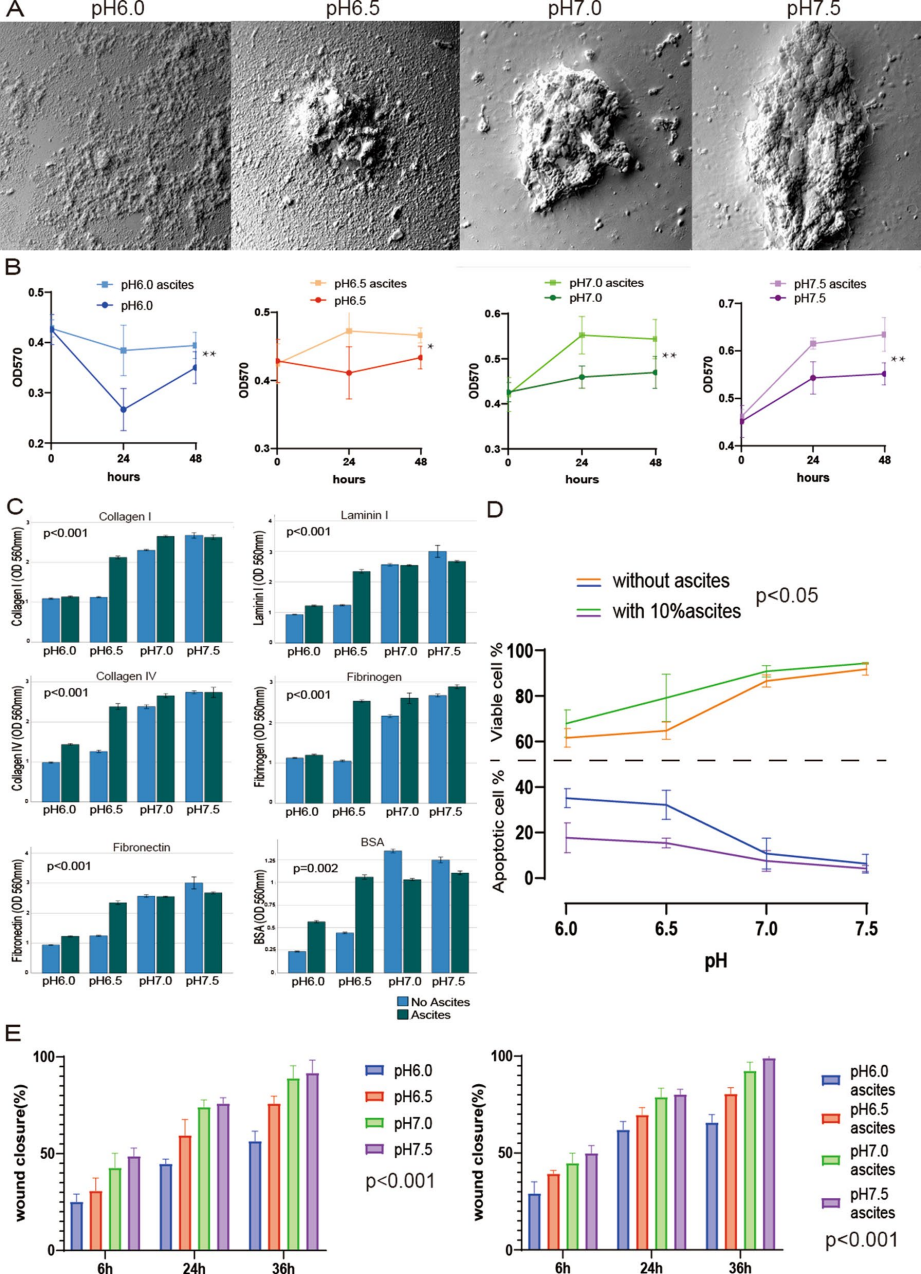
Malignant ascites help cancer cells resist the inhibition of pH changes despite acidic extracellular environment. **A** Scanning electron microscopy (570X): Ovarian cancer spheroids showed barely any growth in the lower pH group and rapid growth in the slightly alkaline environment (pH range: 6.0, 6.5, 7.0, 7.5). **B** MTT assay: Malignant ascites increased the metabolism of tumor cells in different pH environments (n = 6, * < 0.05, ** < 0.01). **C** Adhesion assay: The adhesion of ovarian cancer cells to collagen I, laminin I, collagen IV, fibrinogen, fibronectin, and BSA was inhibited by the acidic environment (n = 3, *p* < 0.001). Furthermore, malignant ascites at pH 6.5 increased the adhesion of cancer cells (*p* < 0.05). **D** Flow cytometry: Apoptotic cell ratio decreases with increasing pH (n = 3, *p* < 0.05). Malignant ascites decreases the rate of apoptotic cells at different pH, especially in an acidic environment. **E** Scratch assay: The acidic environment inhibited the migration ability of ovarian cancer cells (n = 3, *p* < 0.001). Malignant ascites increased the migration of cancer cells

We found that the growth of 3D ovarian cancer spheroids was significantly inhibited in the acidic environment (Additional file 1: Fig. S1), and some broken cell fragments could be found on the surface of 3D ovarian cancer spheroids in pH 6.0 and pH 6.5 groups (Fig. 1A), indicating that the highly acidic extracellular environment could effectively inhibit the growth of cancer spheroids. We investigated the effects of different pH values (6.0, 6.5, 7.0, 7.5) on cancer cell marker functions via cellular experiments, including cell metabolic activity (Fig. 1B), adhesion (Fig. 1C), anti-apoptosis (Fig. 1D, Additional file 1: Fig. S2) and migration ability (Fig. 1E). The results showed that all indicators of cancer cell functions were suppressed in an acidic environment (*p* < 0.001). Intriguingly, when we added 10% of malignant ovarian cancer ascites to co-culture the cancer cells, malignant ascites helped the ovarian cancer cells to resist the inhibitory effect of pH alteration on various functions (*p* < 0.05).

### Differential genes between cancer cells in ascites vs. primary and metastatic cancer cells focus on metabolic and immune pathways

**Fig. 2:**
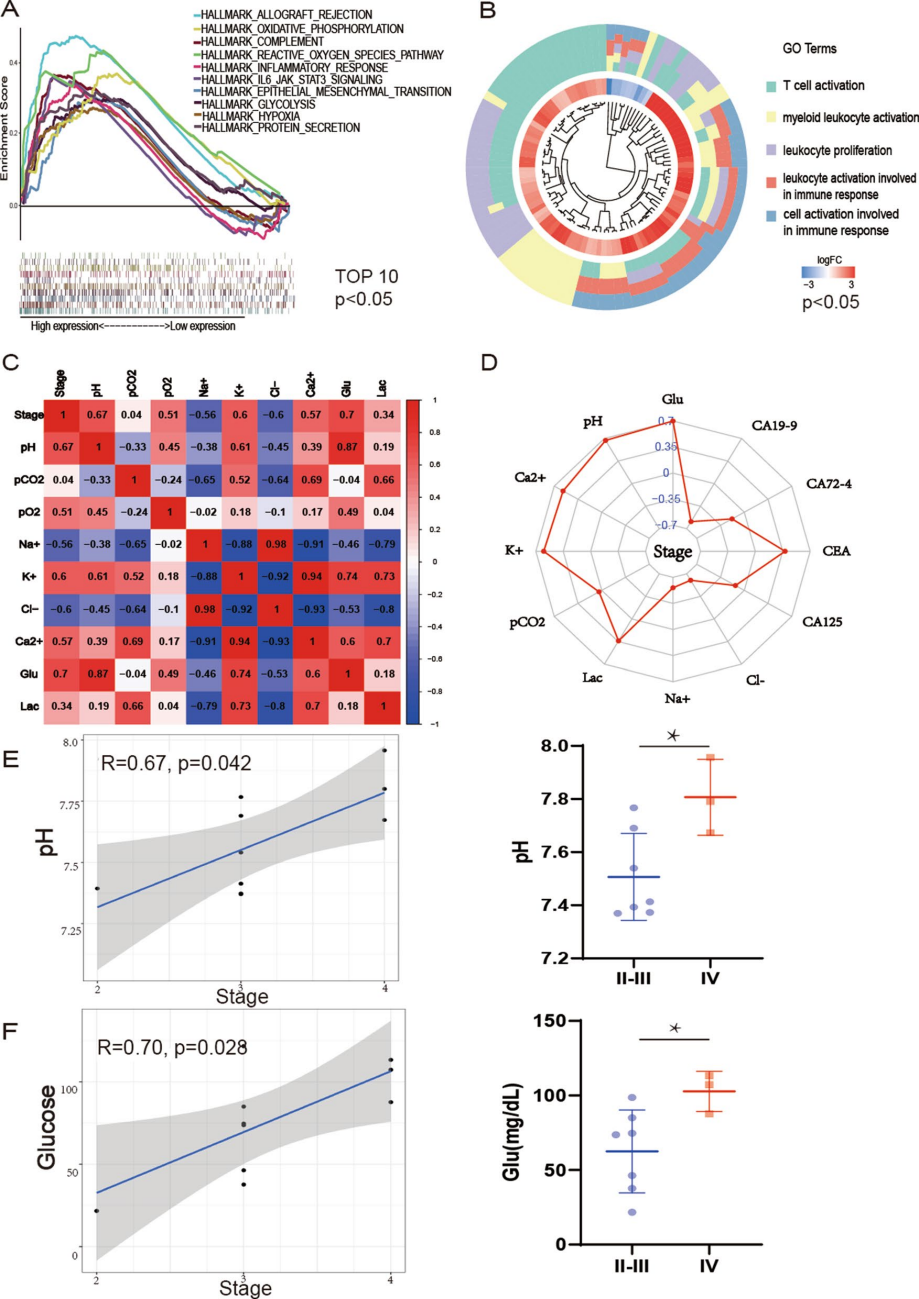
Bioinformatics analysis of ascites ovarian cancer cells and primary or metastatic cancer cells. **A** Gene set enrichment analysis (GSEA) analysis: The first ten hallmark gene sets of ascites cells and primary cells (*p* < 0.05). **B** The circle plots show Gene ontology (GO) pathway enrichment data for differential genes in ascites cells (*p* < 0.05). **C** Correlation of physical–chemical parameters of ascites. **D** Radar plot shows the correlation of ascites parameters and tumor markers with patient stages. **E** Spearman plot and box plot of pH and stages. **F** Spearman plot and box plot of glucose and stages (* < 0.05)

Through cellular experiments, we found that malignant ascites increased the biological properties of cancer cells, then, we investigated the effect of malignant ascites on cancer cells at the genetic level. We analyzed the GSE73065 database [17] for differential gene expression between ascites and primary cells (log_2_|FC|> 1, *p* < 0.05). Compared to primary cancer cells, ascites cells expressed 517 upregulated genes and 222 down-regulated genes. GSEA revealed that ascites cell genes were enriched in metabolic pathways like glycolysis and oxidative phosphorylation and immune responses like complement and inflammatory response compared to primary cells (Fig. 2A) (*p* < 0.05). Gene ontology GO pathway enrichment analysis explores the potential biological functions of DEGs (Fig. 2B). As ascites cancer cells genes mainly focused on metabolism and immunity changes, we further investigated the physicochemical indicators, metabolites, and cytokine in the collected ascites of malignant ovarian cancer.We used a blood-gas analyzer to measure pH, electrolytes, partial pressure distribution, and metabolites of malignant ovarian ascites. In the study of ascites, we used the "corrplot" package in R language to examine the relationship between these parameters. The stage of patients was positively correlated with pH, K^+^, Ca^2+^, and glucose (R > 0.5) of ascites and negative with Na^+^ and Cl^−^ (R < -0.5) (Fig. 2C). The correlation coefficient between those parameters in ascites and patients stage was even higher than that between patient stage and tumor markers in serum (Fig. 2D). The pH values of malignant ovarian ascites were higher in stage IV than in stage II-III patients which further supported the findings of our cellular experiments. Spearman analysis showed a positive and statistically significant correlation between pH and stage of patients (R = 0.67, *p* < 0.05) (Fig. 2E). We also observed that the glucose concentration in ascites was higher in stage IV patients than in stage II-III and was positively correlated with patient stage (R = 0.69, *p* < 0.05) (Fig. 2F). K^+^, Ca^2+^, and lactate were higher in stage IV, but pCO_2_, Na^+^, and Cl^−^ were lower (Additional file 1: Table S2).

### Ascites polar and lipid metabolomics

**Fig. 3:**
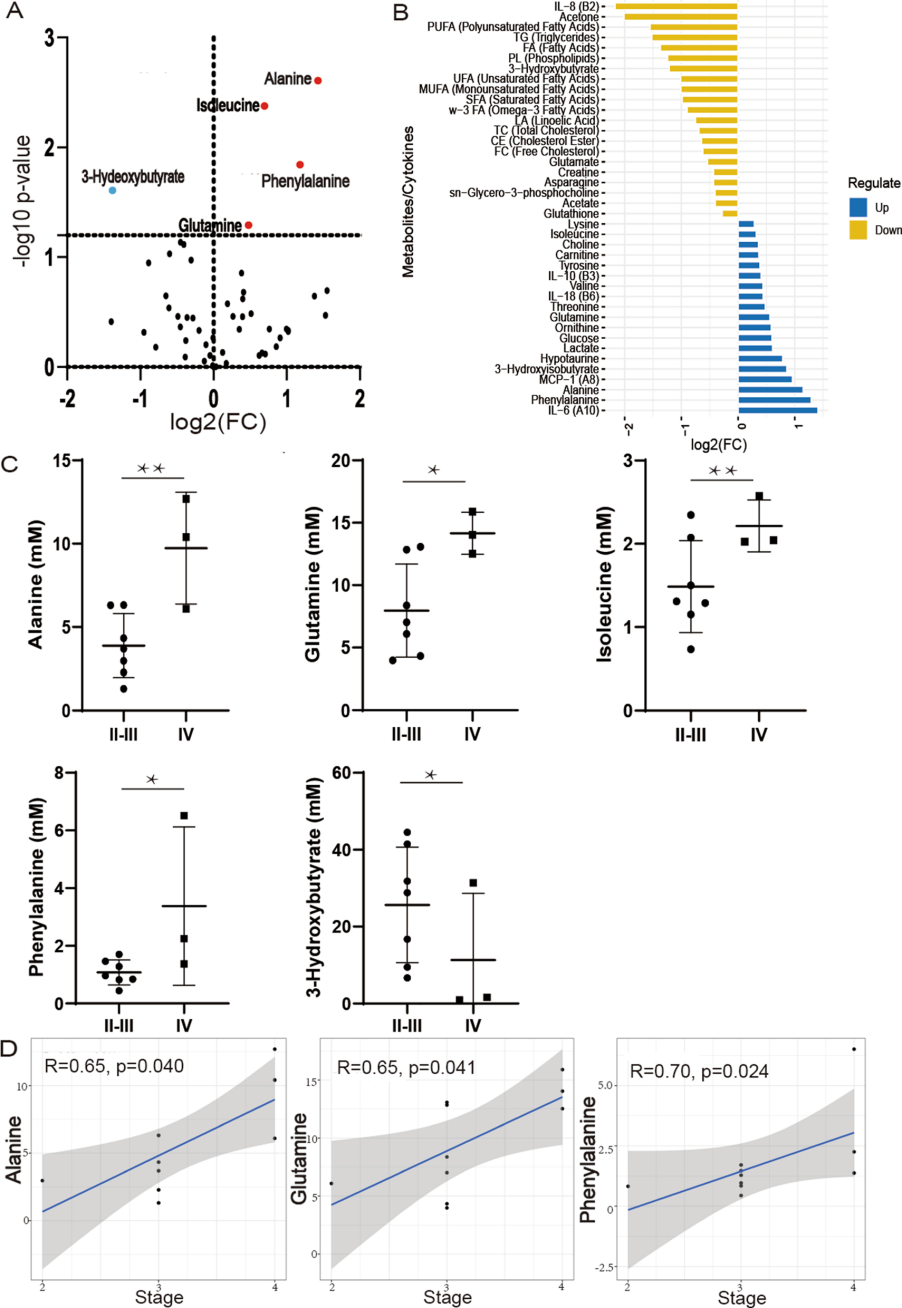
Polar and lipid metabolites levels in ovarian cancer ascites of stages II-III vs IV. **A** Volcano plot indicating statistically significant polar and lipid metabolites changes between the tumor stages: red and blue plots mean up and down regular in stage IV. **B** Bar plot of fold change (FC) of pola and lipid metabolites and cytokines, blue and yellow means upregulation and downregulation in stage IV. **C** Individual metabolite dot plots showing alanine, glutamine, isoleucine, phenylalanine,valine and 3-hydroxybutyrate concentration changes between tumor stages II-III and IV (* < 0.05, ** < 0.01). **D** Spearman plot of alanine, glutamine and phenylalanine with stages (*p* < 0.05)

Based on the FC > 1.2, *p* < 0.05, we identified that alanine, isoleucine, phenylalanine, and glutamine were upregulated, while 3-hydroxybutyrate was downregulated in stage IV compared to II-III (Fig. 3A) (Additional file 1: Table S1). Compared to stage II-III with FC > 1.2, 19 metabolites and cytokines were upregulated and 21 down-regulated in stage IV (Fig. 3B). T-test analysis was performed according to the stage of ovarian cancer patients. lanine, glutamine, isoleucine and phenylalanine were significantly upregulated in stage IV, and 3-hydroxybutyrate were significant downregulated in stage IV (Fig. 3C). Among them, alanine, glutamine and phenylalanine were positively associated with the stage of the disease (*p* < 0.05) (Fig. 3D).

**Fig. 4:**
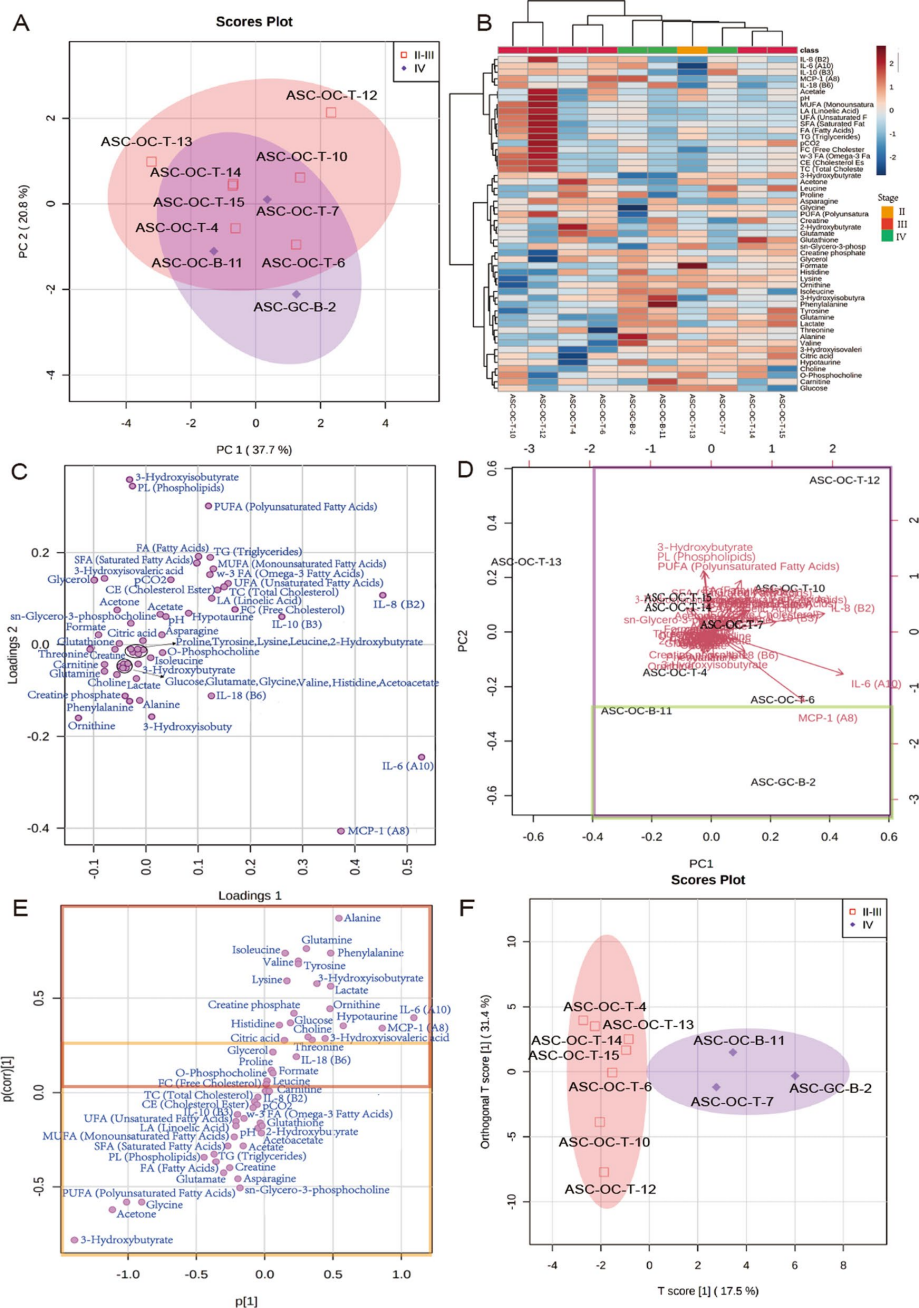
Metabolomics of multivariate statistical analysis on ovarian cancer ascites stages II-IV. **A** Principal component analysis (PCA) scores plot of OCs. **B** Heatmap with all the parameters based on stages II-III versus IV. **C** PCA loadings plot of illustrating features that drive principal component separation. **D** PCA biplot, green and purple boxes indicate the involvement of in other organs and omentum majus, respectively. **E** Orthogonal orthogonal partial least-squares discrimination analysis (OPLS-DA) -loadings plot, orange box indicates metabolite involvement in omentum, while red box indicates involvement in other organs. **F** OPLS-scores plot of OCs illustrates clear II-III and IV group separation

PCA and OPLS were further performed to show how the OCs were clustered based by their metabolic phenotypes. In the PCA-score plot, the OCs with stage II-IV had a similar metabolic phenotype; ASC-OC-T-7 with IV was clustered with the OCs with II-III (Fig. 4A). In other words, the metabolic phenotype of the OC with II-IV varied due to certain involvements, further confirmed by the heatmap (Fig. 4B). Nevertheless, the OPLS score plot showed that the metabolic phenotype of the OCs was different in each clinical stage (Fig. 4F). Interleukin 6 (IL-6), Interleukin 8 (IL-8), Monocyte chemoattractant protein-1 (MCP-1), polyunsaturated fatty acid (PUFA), 3-hydroxybutyrate, glycine, and acetone were discriminated concerning other parameters in the PCA-loading plot (Fig. 4C). The PCA biplot was used to identify the distinct metabolic phenotype in each patient (Fig. 4D). Based on the PCA biplot, ASC-OC-B-11 and ASC-GC-B-2 exhibited other organ metastases for their tumor progression. ASC-OC-T-13 was the only positive sample for ascites without peritoneal metastasis (PM) and showed the highest formate concentration (Fig. 4B).

### Survival of OC patients could be affected by dysregulated polar, lipid metabolism and inflammation

**Fig. 5:**
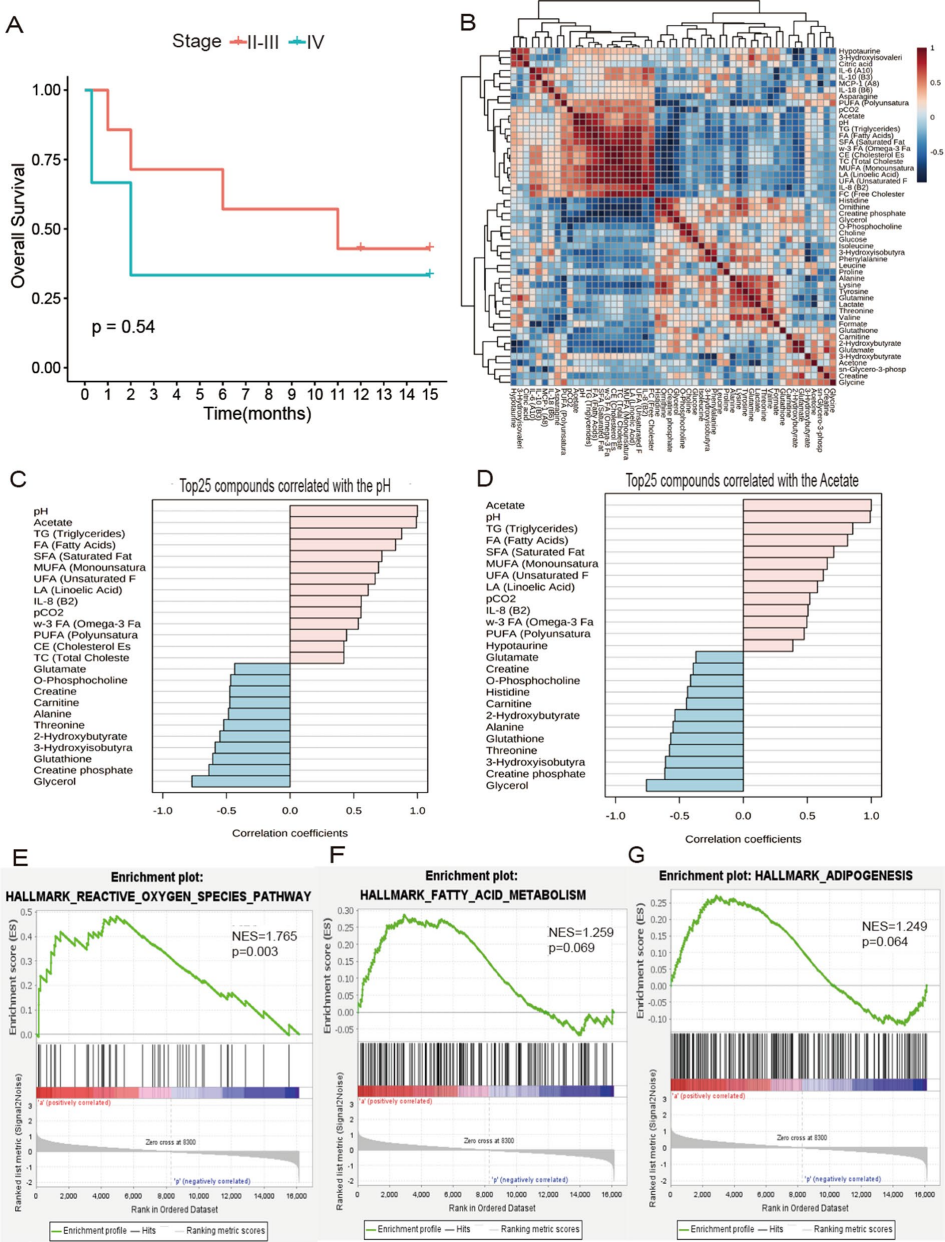
Dysregulated polar, lipid metabolism and inflammation could impact the survival of OC patients. **A** Patients Kaplan–Meier curve. **B** Correlation heatmap of all the selected parameters. Pattern hunter correlation analysis of **C** pH and **D** acetate. Gene enrichment plots of ascites cancer cells on **E** reactive oxygen species pathway, **F** fatty acid metabolism and **G** adipogenesis

Patients with stage IV have a shorter overall survival time, as indicated by Kaplan–Meier curve (Fig. 5A). pH was positively correlated with acetate, branched-chain amino acids (BCCAs), asparagine, and sn-glycerol-3-phosphocholine, and negatively correlated with creatine phosphate, as shown by correlation heat map (Fig. 5B). Pattern hunter correlation analysis shown, pH positively correlated with acetate, and acetate positively correlated with lipid compounds (Fig. 5C). Moreover, GSEA showed that ascites cancer cells were enriched in reactive oxygen species pathway (NES = 1.765, *p* = 0.003), fatty acid metabolism (NES = 1.259, *p* = 0.069), and adipogenesis (NES = 1.249, *p* = 0.064) (Fig. 5D).

**Fig. 6:**
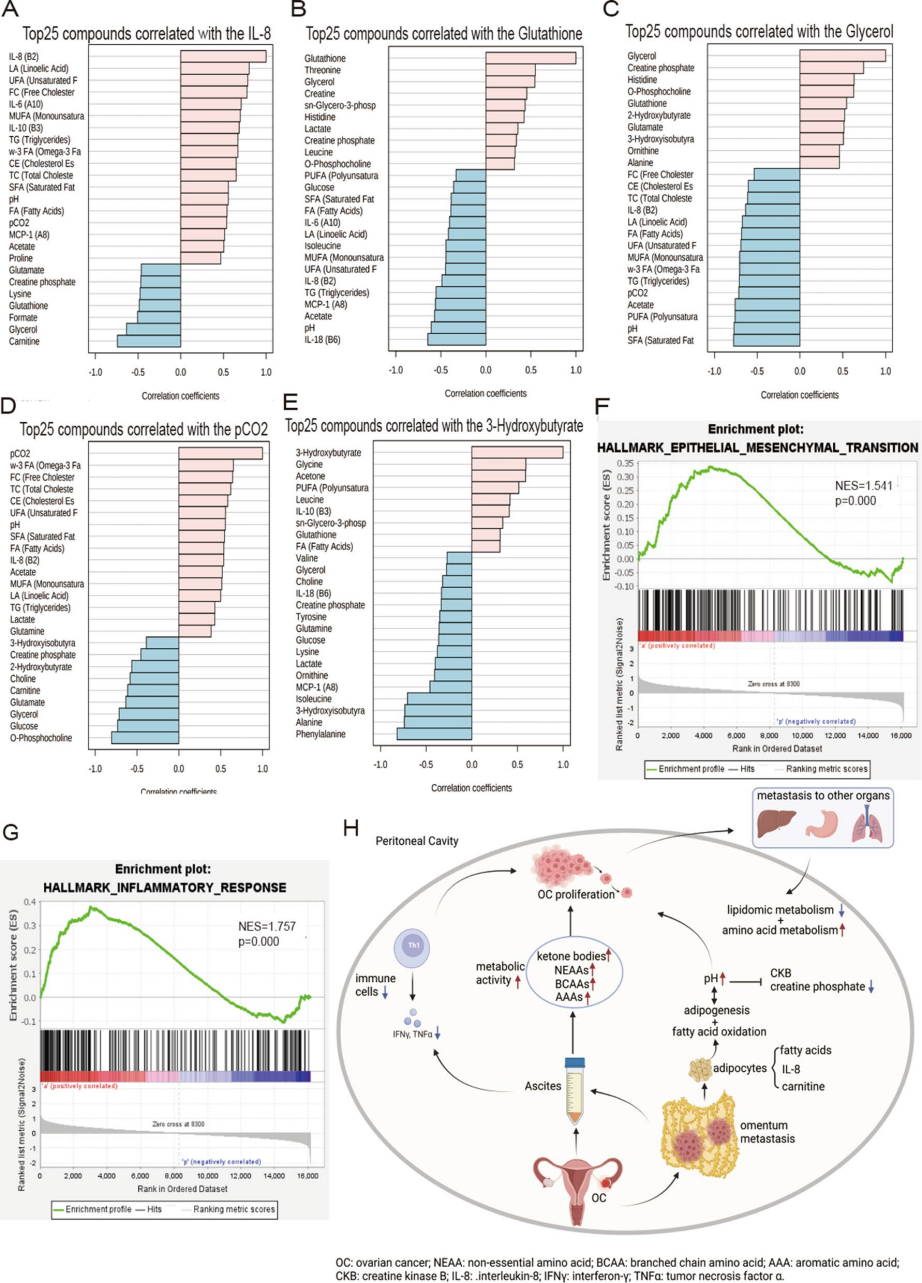
A nexus of dysregulated metabolic pathways, inflammation, and cell transitions in OC patients. Pattern hunter correlation analysis of **A** IL-8, **B** glutathione, **C** glycerol, **D** pCO2, **E** 3-Hydroxybutyrate. Ascites cancer cells genes enrichment plots of **F** epithelial-mesenchymal transition and **G** inflammatory response. **H** Graphical abstract of metabolites, cytokines and pH interaction in ascites and their association with cellular proliferation and organ metastasis in ovarian cancer

Based on Pearson's r test correlation analysis, IL-8 was positively correlated with lipid metabolites and negatively with carnitine and glycerol (Fig. 6A). Glutathione was positively correlated with threonine and glycerol and negatively with IL-8 and pH (Fig. 6B). Glycerol positively correlated with creatine phosphate and negatively with pH and lipid metabolites (Fig. 6C). pCO_2_ was positively correlated with lipid metabolites and negatively correlated with o-phosphocholine, glucose, and glycerol (Fig. 6D). 3-Hydroxybutyrate was positively correlated with glycine and acetone and mostly negatively correlated with phenylalanine and alanine (Fig. 6E). Genes of ascites cancer cells were enriched in epithelial-mesenchymal-transition (NES = 1.541, *p* < 0.001) (Fig. 6F) and inflammatory response (NES = 1.757, *p* < 0.001) (Fig. 6G). Finally, we produced a graphical abstract to present metabolites, cytokines, and pH interactions in ascites and their association with ovarian cancer cellular proliferation and organ metastasis (Fig. 6H).

## Discussion

Malignant ascites is strongly associated with multifactorial pathophysiology. As malignant ascites develops, tumor vessel permeability increases, and peritoneal fluid is produced and released in higher orders. It has been demonstrated in various studies that small vessels are more permeable due increased peritoneal parietal revascularization and glycoprotein production [19]. Another factor for the production of ascites is invasive lymphatic channels alongside micro- and macro-invasions [20]. Malignant ascites plays a significant role in the intraperitoneal dissemination in ovarian cancer. Malignant ascites generates an immune-suppressive milieu [21], stimulates tissue vascularization through the release of proangiogenic proteins [7], induces epithelial-mesenchymal transition (EMT), encourages the trans-mesothelial invasion of cancer cells [22], and induces premature senescence of normal peritoneal mesothelial cells (PMCs), forcing them to develop a cancer-promoting phenotype [23].

### Dysregulation of pH in malignant ascites of OC patients and its correlation with polar and lipid metabolites

Dysregulation of cellular pH is frequently observed in solid tumors, and acidic extracellular pH (pHe) of several types of tumor tissues has been reported [24, 25]. According to our experiments, the pH of ascites differs from the perception that extracellular fluid is acidic; mean pH was 7.6 ± 0.2. Even though decreasing the pH of cell culture medium can suppress the characteristics of tumor cells, such as metabolic activity, adhesion, anti-apoptosis, and migratory ability, 10% of malignant ascites showed to prevent tumor cells from being affected by acidic pH. Enrichment analysis of differential genes between ascites and primary cancer cells revealed that genes of ascites cells were mainly enriched in metabolic and immune pathways. Thus, ascites pH may affect the metabolism or immunity of cancer cells. According to pattern analysis, the pH of ascites samples was alkaline due to high acetate levels, the conjugate base of a weak acid. High acetate levels can also be seen as an indicator of fatty acid synthesis, as we saw a positive correlation between acetate and fatty acids. Accumulation of acetate has been observed when cancer cells meet nutritional challenges, it becomes a major metabolic source. Some studies have shown that ovarian cancer cells exhibit greater aggressiveness in the ascites microenvironment through reprogramming of lipid metabolism [26]. High lipid metabolic activity was found when ovarian cancer cells were cultured in the ascites microenvironment, suggesting that adipocytes may serve as an energy source for cancer cells [26]. As a result of hypermetabolism, glucose is taken up by cells, and incomplete metabolism leads to pyruvate turnover to acetate, which is then released into the extracellular space [27]. Two mechanisms are involved in the spillover of acetate from pyruvate, i) oxidative decarboxylation mediated by ROS and ii) incomplete oxidation mediated by ketoacid dehydrogenase (kDH) induced by thiamine and glutathione [28]. Other research shows that ovarian cancer cells can induce adipocyte lipolysis, and adaptations to lipid metabolism allow ovarian cancer cells to thrive on lipids accumulated from adipocytes [29]. Therefore, the correlation between pH, acetate, and lipids may indicate that malignant ovarian cancer cells causing ascites may be cross-linked to the omentum majus. In the omentum, fatty acid synthesis, fatty acid oxidation and subsequent ketogenesis are involved in the formation of ascites, thus altering the normal metabolism to a more carcinogenic phenotype.

### The tumor stage of OC patients can be characterized by ascites changes in alanine, glutamine, isoleucine, phenylalanine, and 3-hydroxybutyrate metabolism

Alanine, glutamine, and isoleucine, a BBCA, were significantly higher in OC ascites with stage IV. A significant increase in alanine concentrations in stage IV ovarian cancer ascites supports the theory that tumor malignancy is associated with an increased glycolytic flux, as well as the need for increased protein synthesis in tumors [30]. Glutamate oxidation is believed to be a major source of respiratory energy for cancer cells [31]. It has been shown that glutamine starvation of cancer cells has been effective in tumor therapy [32]. Many studies have found that cancer upregulates high-affinity glutamine transporters [33]. One primary example is the alanine-serine-cysteine transporter 2 (ASCT2), carrying neutral amino acid (AA) such as alanine serine, cysteine, glutamine, and asparagine, which was shown to play a central role in maintaining glutamine homeostasis of Myc-driven cancer cells [34, 35]. In vitro, ASCT2 inhibitor (L-g-glutamyl-p-nitroanilide (GPNA)) can inhibit glutamine uptake and mTOR activation, which regulates protein translation, cell growth, and autophagy [36]. High ASCT2 expression is observed in the cancer cells to fuel their glutamine addiction because glutamine is a central hub in non-essential amino acid (NEAA) metabolism [37, 38]. Glutamine is required to produce glutathione that removes ROS and rescues the cancer cells from oxidative stress-induced apoptosis since ROS is produced by cancer cells due to an increased metabolic rate, gene mutation, and relative hypoxia [39]. Moreover, BCAAs are utilized to synthesize glutamate and glutathione and lead to therapeutic resistances following overexpression of SLC7A5, a BCAA transporter observed in pancreatic, colorectal, gastric, and OC cells [40–42]. Therefore, OC patients with stage IV may be highly Myc-driven and then overexpressed ASCT2 and SLC7A5 to promote glutamine homeostasis. A further study shows that Myc mRNA expression was significantly correlated with clinical stages [43]. MS-based metabolomics on serum of OCs with grade I-IV confirms a significantly higher alanine concentration in OCs than in healthy controls [44]. Furthermore, SLC7A5 overexpression is significantly associated with Myc expression and elevated in high-grade serous ovarian cancer with grade III-IV concerning the normal tissues [45].

In our study, phenylalanine concentration was significantly higher in the OC malignant ascites with stage IV. It has been reported that serum phenylalanine concentrations are higher in patients with ovarian cancer [46]. There were significant correlations between phenylalanine, phenylalanine/ tyrosine, and immune activation markers. In patients suffering from chronic conditions with an immune activation and inflammation background, moderately elevated phenylalanine levels and phenylalanine to tyrosine ratios (Phe/Tyr) have been observed, similar to cancer [46]. These findings suggest that phenylalanine levels in patients with moderate hyperphenylalaninemia may be elevated due to an oxidizing milieu produced by chronic immune responses [47]. Furthermore, it has been reported that phenylalanine metabolism is involved in the suppression of T-cell immune responses and that phenylalanine and its metabolism have a regulatory effect on T-cell proliferation and activation and subsequent immune responses [48].

3-hydroxybutyrate was significantly higher in OC with stage II-III. This metabolite has also been elevated in the serum of epithelial OC patients with (stages I and II) [49], implicating a positive correlation between 3-hydroxybutyrate and the clinical stage of OCs. Additionally, 3-hydroxybutyrate is a biomarker for fatty acid oxidation and ketone metabolism [50], further indicating the reverse Warburg effect and omentum majus involvement rate.

### Correlation of polar and lipid metabolites, cytokines/chemokines, and physical–chemical parameters observed in malignant ascites of OC patients and their critical roles in the tumor progression

Glutathione was negatively correlated with cytokines (IL-6 & IL-18) and chemokines (IL-8 & MCP-1). Glucose is utilized to regenerate NADPH in the PPP to enhance glutathione regeneration [51], as observed in Pearson's r test pattern analysis for glutathione. The negative correlation may also indicate higher glutathione consumption and suppressed immunosurveillance in the OCs. In this report, there was a significant negative correlation between IL-18, MCP-1, and glutathione, implicating direct anti-tumor immunity of Interferon-γ (IFN-γ) producing T helper type 1 (Th1) cells. In response to ROS produced by cancers, the innate immune system is activated, where macrophages and dendritic cells produce proinflammatory cytokines such as tumor necrosis factor (TNF-α) and Interleukin-1β (IL-1β) to enhance adaptive immunity [51–53]. In other words, anti-tumor immunity generates ROS, which requires glutathione to prevent oxidative stress-induced cellular damage [54, 55]. Additionally, it is shown that endogenous glutathione enhances the innate immune system [56]. However, anti-tumor immune cells undergo cell dysfunction and apoptosis due to lower antioxidant capacity in TME than in cancers [57–59]. It is why the negative correlation between proinflammatory cytokines and chemokines, MCP-1 as an indicator of a proinflammatory state [60] were observed, and interferons (IFNs) and TNF-α were almost undetected in the OCs.

Carnitine was negatively correlated with all the cytokines/chemokines except IL-18. IL-8 was only significantly negatively correlated with carnitine and glycerol, IL-8 significantly positively correlated with acetate. The negative correlation may reflect omentum involvement since all the OCs exhibited PM except ASC-OC-T-13. Fatty acid oxidation is an alternative way to produce ATP. Carnitine plays hereby a critical role as those fatty acids are transported as acyl-carnitine into the mitochondria, where fatty acids are oxidized [61]. A study shows carnitine palmitoyl transferase (CPT1) overexpression took place in OC cell lines and primary ovarian serous carcinoma, and its overexpression was correlated with poor survival in OCs [62]. Another study has revealed that OC cells took exogenous fatty acids from the omentum for their tumorigenesis and metastasis [29, 63]. Adipocytes facilitate the metastasis of OC cells by secreting adipokine (IL-6, IL-8, and MCP-1), and IL-8 is the one activating adipocyte together with fatty acid-binding protein 4 (FABP4) to provide fatty acids in OCs microenvironment [29, 64]. The negative correlation of glycerol with IL-8 may then reflect lipolysis where triglycerides were broken down into fatty acids and glycerol in the OCs further confirmed by the negative correlation glycerol and lipid metabolite species. A recent study shows glycerol-3-phosphate acyltransferase 1 was overexpressed to enhance their adhesion and migration and associated with poor survival in OCs [65].

The positive correlation between acetate and fatty acids may reflect lipid-depleted-metabolic stress in the OCs since the accumulation of acetate has been observed to benefit breast, ovarian, and lung cancers where acetyl-CoA synthetases (ACS) are highly expressed to utilize acetate to form acetyl CoA [66]. Visceral adipose tissue (VAT)-associated CD4^+^ Tregs are found in the omentum, producing high levels of IL-10 to constitute Treg subpopulation and then suppressing the anti-tumor immune system [67, 68]. In Pearson's r test with pattern hunter, 3-hydroxybutyrate positive correlated with BCAAs, aromatic AAs, and glutathione were observed, pointing that high-rate of fatty acid oxidation followed by ketogenesis for ATP production resulted in an elevated level of ROS intracellularly in the OCs. Furthermore, the positive correlation of 3-hydroxybutyrate with PUFA was higher than with other lipid metabolite species, implicating PUFA-mediated inhibition of T cell immune response by modified lipid rafts [69]. PUFA-mediated tumor progression is also observed in colorectal cancer and only endometrial cancer with reoccurrence [70, 71]. Such findings explain why ketone bodies were negatively correlated with MCP-1 and IL-18 and positively correlated with IL-10. Hence, the metabolite phenotype revealed the magnitude of proliferative progression of the OCs and the omentum majus as a new cell homing organ for tumors.

The negative correlation may be reflective of omentum involvement in glutamine synthesis for tumor progression in which alanine and BCAAS contribute to glutamine homeostasis and glutathione production. In pancreatic cancers, adipocytes downregulate glutaminase to secrete and transfer glutamine under glutamine-deprived conditions, and the glutamines may reflect catabolism of lipid stores [72]. Finally, the negative correlation with creatine phosphate could also implicate that OC cells obtained their ATP mostly via fatty acid oxidation once they metastasized in the omentum. Overexpression of creatine kinase B (CKB) takes place in breast, colorectal, and OCs in response to acute hypoxia exposure [73–75] due to more ATP production demand for proliferation [52, 76, 77], which means hypoxic OC cells can elevate glucose consumption and lactate production, and decreased ROS production by CKB [78]. However, lactate production decreased or had a low negative correlation with pH, indicating less active glycolysis. This may reflect inactivated CKB in the omentum, and OC cells could only acquire ATP via fatty acid oxidation, relying on the adipocytes. Hence, acetate produced by the OC cells and/or the adipocytes made the microenvironment basic at which CKB was inactivated, which may be why CKB is not considered as a prognostic marker for OCs with later clinical stage and/or ascites positive.

### Metabolites, lipids, cytokines/chemokines, and physical–chemical parameters allow for a refined stratification of OC patients suffering from malignant ascites

The PCA and oPLSDA loading plots and PCA biplots showed that each OC patient exhibited a specific carcinogenic phenotype. An overall heatmap was created to see how all the parameters contributed to each OC progression, including clinical stage and TNM classification of the OCs. Here, the OCs with omentum metastasis involvement had a shorter survival time than the OCs without omentum metastasis. Studies have shown that the 5-year overall survival (OS) rates in OCs with omental metastasis were 43.4% [79], but the median survival following diagnosis of ascites was only 5.7 months [80]. Therefore, if metabolomics, cytokine, and physicochemical parameters of ascites are analyzed and finely stratified, they can serve as future treatment modalities and tailored to target individual patient differences more effectively.

Special attention must be paid to ASC-OC-T-10 and ASC-OC-T-12 patients where large omental metastases called "omental cakes" were observed, as a result of metastasis from the ovary to the omentum. These two OCs had the highest concentration of lipid metabolite species among the OCs. The endogenous production of fatty acids constitutes a source of oncogenic stimuli that feed the malignant progression of tumors [81]. An anabolic/catabolic switch in cancer cells allows the cells to grow rapidly and progress aggressively. Lipolytic enzyme activity, fatty acids, and glycerol are increased in response to metabolic stress. A fatty acid cycling network supports malignancy with heightened lipolysis and lipogenesis in cancer cells [82]. This may explain the poorer prognosis of ASC-OC-T-10 and ASC-OC-T-12, with OS of only 6 and 11 months, respectively.

ASC-OC-B-11 and ASC-GC-B-2 were stage IV and metastasized to the pleura and stomach. These two OCs had higher concentrations of cytokines (MCP-1, IL-6, and IL-18) among the OCs. Except for ASC-OC-T-13, all patients had omental metastases, but the metabolic phenotypes differed based on fat metabolism and organ metastases. Interestingly, according to the PCA-loading plot, although ASC-OC-T-6 was stage III, the metabolic phenotype was closer to stage IV, ASC-GC-B-2 and ASC-OC-B-11. At the same time, ASC-OC-T-7 was stage IV, but it was closer to stage III, ASC-OC-T-4 and ASC-OC-T-14. Furthermore, this metabolic phenotype could explain how each OC progressed, which was relevant to survival rates. They had a closer prognosis, with ASC-GC-B-2, ASC-OC-B-11, and ASC-OC-T-6 patients having died with overall survival of only 0.3, 2, and 2 months, whereas ASC-OC-T-7, ASC-OC-T-4, and ASC-OC-T-14 patients still alive with overall survival were up to 15, 15, and 12 months. For ASC-OC-T-15, although this metabolic phenotype of the patient was closer to ASC-OC-T-4 and ASC-OC-T-14, the survival was shorter. It may be because this patient was initially diagnosed with ovarian cancer in 2017, and this hospitalization was due to a recurrence of ovarian cancer.

ASC-OC-T-13 with stage II exhibited different metabolic and cytokine profiles that formate and cytokines were found highest and lowest, respectively. It may reflect that high mitochondrial glycine-formate metabolism was crucial for the invasion, which is supported by the production of serine, something which has been observed before in glioblastoma multiforme cell lines [83].

## Conclusion

In this report, polar and lipid metabolites of malignant ascites from OC patients were analyzed and quantified by ^1^H-NMR spectroscopy-based metabolomics, with a 13-plex cytokines/chemokines panel and physical–chemical parameters. Each OC patient showed a unique metabolic and cytokine/chemokine profile that could further explain clinical stages and TNM classification. Indeed, NMR spectroscopy-based metabolomics would further stratify ovarian cancer patients according to their different metabolic phenotypes. The data will assist in determining the diagnosis and prognosis of patients with OC as well as their treatment targets. Analyzing metabolic preferences in malignant ascites is informative for discovering new paradigms for metabolic regulation in proliferating cancer cells.

## Supplementary Information

**Additional file 1: Figure S1. **An acidic extracellular environment inhibits the proliferation of 3D spheroids. **Figure S2.** Flow cytometric analysis of the effect of pH on apoptosis of OAW42 cells in 10% malignant ovarian ascites at different pHs. **Table S1.** Fold changes of polar metabolites on ovarian cancers with II-III vs. IV. **Table S2.** Full matrix of all obtained molecular and physical-chemical ascites parameters of stages II-III and IV. **Table S3.** Patient Survival Time Information. **Table S4.** Patient peritoneal metastasis information.

## Data Availability

The data presented is partially obtained by analyzing already publicly available data. The datasets used and analyzed during the current study are available from the corresponding author on reasonable request. ^1^H-NMR spectra data and concentration tables of cytokines are available upon request.

## Abbreviations

OC: Ovarian cancer
FIGO: International federation of gynecology and obstetrics
CDK1: Cyclin-dependent kinase 1
HIF-1⍺: Hypoxia-inducible factor 1⍺
ECM: Extracellular matrix
PPP: Pentose phosphate pathway
ATP: Adenosine triphosphate
ROS: Reactive oxygen species
CAFs: Cancer-associated fibroblasts
NMR: Nuclear magnetic resonance
SEM: Scanning electron microscopy
MTT: 3-(4,5-Dimethylthiazol-2-yl)-2,5-diphenyltetrazolium bromide
BSA: Bovine serum albumin
GEO: Gene expression omnibus
GSEA: Gene set enrichment analysis
NES: Normalized enrichment score
GO: Gene ontology
CPMG: Carr-Purcell-Meiboom-Gill pulse programs
MTBE: Tert-butyl methyl ether
TSP: 3-(trimethylsilyl)propionic-2,2,3,3 acid sodium salt
NOESY1D: Nuclear Overhauser Spectroscopy
SNR: Signal-to-noise ratio
GPLC: High performance liquid chromatography
TMS: Tetramethylsilane
PQN: Probabilistic quotient normalization
FC: Fold change
PCA: Principle component analysis
OPLS-DA: Orthogonal projections to latent structures discriminant analysis
PUFA: Polyunsaturated fatty acid
PM: Peritoneal metastasis
BCCAs: Branched-chain amino acids
EMT: Epithelial-mesenchymal transition
PMCs: Peritoneal mesothelial cells
ASCT2: Alanine-serine-cysteine transporter 2
GPNA: L-g-glutamyl-p-nitroanilide
NEAA: Non-essential amino acid
CPT1: Carnitine palmitoyl transferase 1
FABP4: Fatty acid-binding protein 4
ACS: Acetyl-CoA synthetases
CKB: Creatine kinase B
VAT: Visceral adipose tissue
OS: Overall survival
