# Supplementary material for: Acidic ascites inhibits ovarian cancer cell proliferation and correlates with the metabolomic, lipidomic and inflammatory phenotype of human patients
Source: J Transl Med. 2022 Dec 12;20:581. doi: 10.1186/s12967-022-03763-3 (PMC9743551; doi:10.1186/s12967-022-03763-3)
Supplement: Supplementary file 1 — Additional file 1: Figure S1. An acidic extracellular environment inhibits the proliferation of 3D spheroids. Figure S2. Flow cytometric analysis of the effect of pH on apoptosis of OAW42 cells in 10% malignant ovarian ascites at different pHs. Table S1. Fold changes of polar metabolites on ovarian cancers with II-III vs. IV. Table S2. Full matrix of all obtained molecular and physical-chemical ascites parameters of stages II-III and IV. Table S3. Patient Survival Time Information. Table S4. Patient peritoneal metastasis information. [file 12967_2022_3763_MOESM1_ESM.pdf]

Additional File 1

Acidic ascites inhibits ovarian cancer cell proliferation and correlates with the metabolomic, lipidomic and inflammatory phenotype of human patients

Qianlu Yang[1,2], Gyuntae Bae[2], Giorgi Nadiradze[1,3], Arianna Castagna[1,3], Georgy Berezhnoy[2], Laimdota Zizmare[2], Aditi Kulkarni[2], Yogesh Singh[4,5], Frank J. Weinreich[1], Stefan Kommoss[5], Marc A. Reymond[1,3], Christoph Trautwein[2,*]

[1] National Center for Pleura and Peritoneum, NCT South-West Germany, Tübingen, Germany
[2] Department of Preclinical Imaging and Radiopharmacy, Werner Siemens Imaging Center, University Hospital Tübingen, Germany
[3] Department of General and Transplant Surgery, University Hospital Tübingen, Germany
[4] Institute of Medical Genetics and Applied Genomics, University Hospital Tübingen, Germany
[5] Research Institute of Women's Health, Women's Hospital, University Hospital Tübingen, Germany

* Corresponding author name and email address: christoph.trautwein@med.uni-tuebingen.de

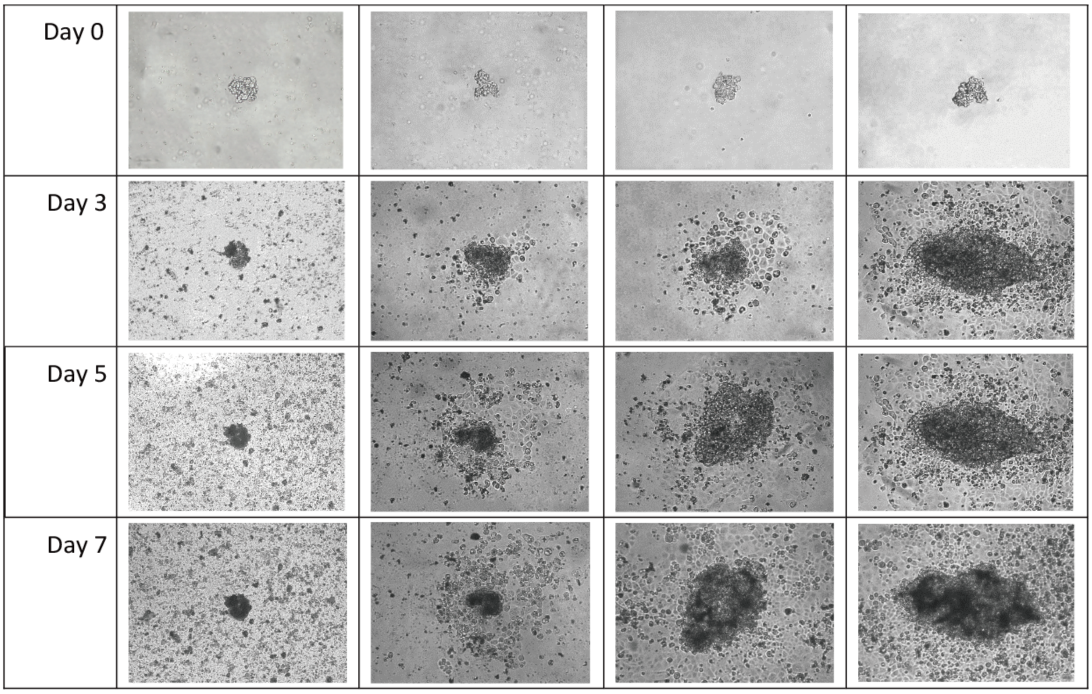

Figure S1. An acidic extracellular environment inhibits the proliferation of 3D spheroids
Light microscopy (10X): Ovarian cancer spheroids showed barely any growth in the lower pH group and rapid growth in the slightly alkaline environment (pH range: 6.0,6.5,7.0,7.5).

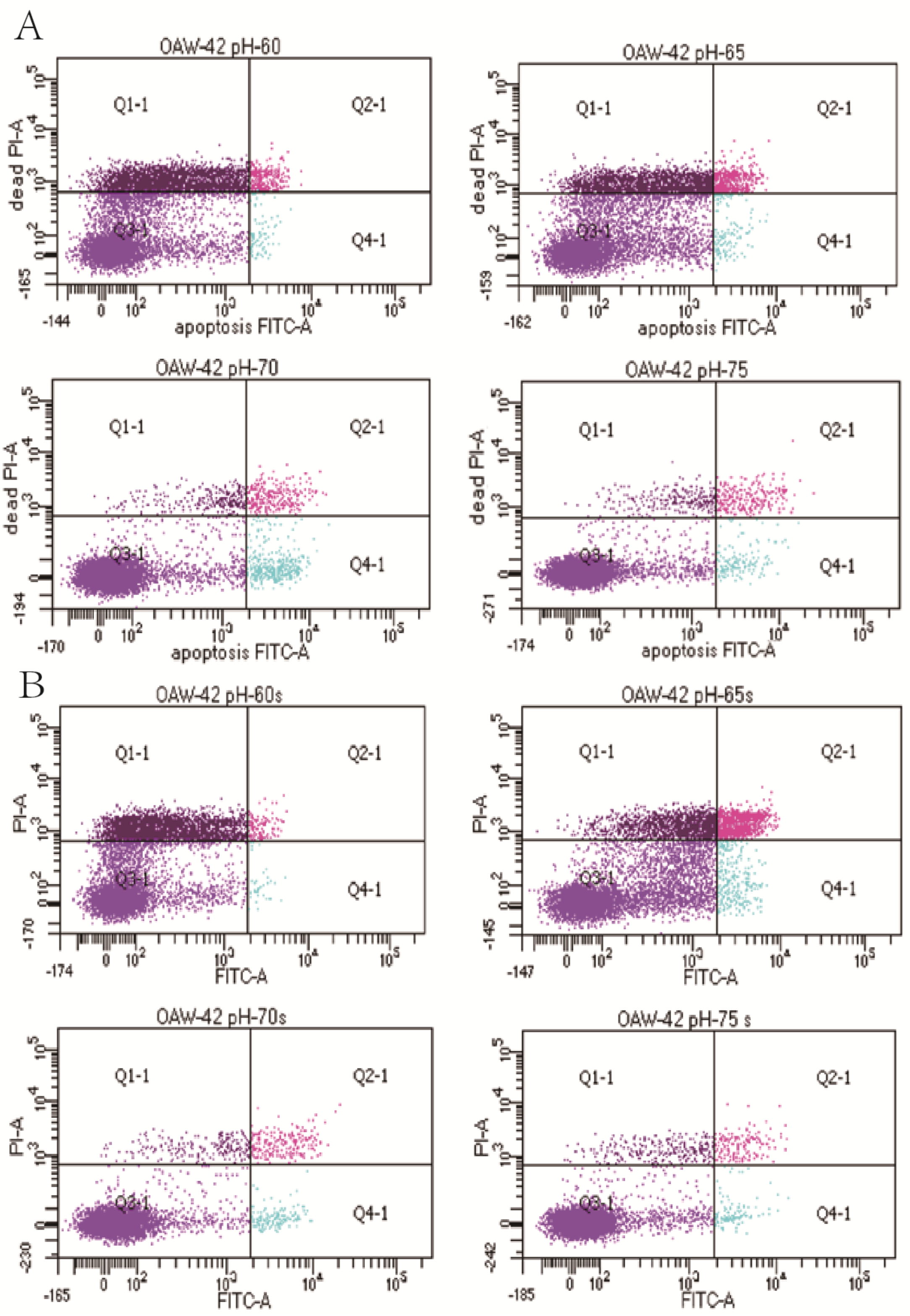

Figure S2. Flow cytometric analysis of the effect of pH on apoptosis of OAW42 cells in 10% malignant ovarian ascites at different pHs

A）Apoptosis rate of OAW45 cells at different pHs (6.0, 6.5, 7.0,7.5). B) Apoptosis rate of OAW45 cells at different pHs with 10% ascites.

Table S1 – Fold changes of polar metabolites on ovarian cancers with II-III vs IV.

	FC	log2(FC)	raw.pval	-log10(p)
Alanine	0.44628	-1.164	0.002351	2.6288
Isoleucine	0.79325	-0.33415	0.0040014	2.3978
Phenylalanine	0.39805	-1.329	0.014645	1.8343
3-Hydroxybutyrate	2.3475	1.2311	0.02715	1.5662
Glutamine	0.66784	-0.58243	0.049762	1.3031

Table S2 – Full matrix of all obtained molecular and physical-chemical ascites parameters of stages II-III and IV.

Parameter	Stage II- III	Stage IV
pH	7.51±0.16	7.78±0.16
pCO2 (mmHg)	22.05±14.56	17.33±2.08
Na+ (mmol/L)	142.00±8.04	136.22±1.35
K+(mmol/L)	3.24±1.61	4.34±0.63
Ca2+(mmol/L)	0.89±0.38	1.12±0.06
Cl-(mmol/L)	121.24±17.67	108.77±5.59
Glu (mg/dL)	65.81±33.54	102.78±13.42
Lac (mmol/L)	1.73±1.36	2.10±0.85
2-Hydroxybutyrate	2.13±1.42	2.22±0.96
3-Hydroxybutyrate	25.64±15.04	11.31±17.39
3-Hydroxyisobutyrate	0.32±0.17	0.68±0.45
3-Hydroxyisovaleric acid	1.91±1.01	1.81±0.66
Acetate	3.29±0.15	3.29±0.21
Acetone	2.69±4.96	0.57±0.72
Alanine	3.87±1.92	9.73±3.35
Asparagine	1.45±0.62	1.37±0.81
Carnitine	0.74±0.41	1.15±0.87
Choline	0.22±0.19	0.32±0.11
Citric acid	0.74±0.34	0.98±0.19
Creatine	1.34±0.83	1.21±1.21
Creatine phosphate	1.06±0.56	1.44±0.26
Formate	0.86±0.57	0.96±0.22
Glucose	75.72±33.67	138.16±47.17
Glutamate	4.60±3.54	3.75±3.09
Glutamine	7.95±3.73	14.15±1.68
Glutathione	0.45±0.24	0.41±0.07
Glycerol	6.23±2.95	7.27±1.72
Glycine	5.69±3.02	8.58±1.96
Histidine	1.41±0.72	1.79±0.28

Hypotaurine	1.13±0.61	1.92±1.86
Isoleucine	1.49±0.55	2.21±0.31
Lactate	38.13±30.99	69.74±35.05
Leucine	3.25±1.59	3.66±0.60
Lysine	3.36±1.43	4.86±0.82
O-Phosphocholine	0.43±0.19	0.482±0.08
Ornithine	1.47±0.44	1.921±0.68
Phenylalanine	1.08±0.43	3.378±2.75
Proline	2.83±1.73	3.358±1.11
Threonine	4.09±3.09	5.09±0.89
Tyrosine	1.10±0.51	1.69±0.18
Valine	5.09±2.11	7.97±0.93
sn-Glycerol-3-phosphocholine	1.34±0.54	1.26±0.65
CE (Cholesterol Ester)	0.39±0.32	0.32±0.07
FA (Fatty Acids)	0.01±0.01	0.006±0.00
FC (Free Cholesterol)	0.29±0.19	0.26±0.13
LA (Linoleic Acid)	0.03±0.02	0.02±0.01
MUFA (Monounsaturated Fatty Acids)	0.01±0.01	0.008±0.00
PL (Phospholipids)	0.32±0.06	0.20±0.02
PUFA (Polyunsaturated Fatty Acids)	0.03±0.02	0.02±0.00
SFA (Saturated Fatty Acids)	0.10±0.07	0.07±0.01
TC (Total Cholesterol)	0.362±0.29	0.29±0.06
TG (Triglycerides)	0.008±0.01	0.004±0.00
UFA (Unsaturated Fatty Acids)	0.04±0.03	0.03±0.01
w-3 FA (Omega-3 Fatty Acids)	0.08±0.06	0.06±0.01
MCP-1 (A8)	840.45±1359.80	1998.66±3120.40
IL-6 (A10)	4510.35±3502.17	15267.12±14619.95
IL-8 (B2)	650.87±1138.10	281.04±297.60
IL-10 (B3)	115.67±76.64	166.62±228.32
IL-18 (B6)	179.14±162.49	322.41±283.25

Table S3 – Patient Survival Time Information.

ID	Survival (Months)	State	ID	Survival (Months)	State
ASC-GC-B-2	0.3	dead	ASC-OC-B-11	2	dead
ASC-OC-T-4	15	alive	ASC-OC-T-12	11	dead
ASC-OC-T-6	2	dead	ASC-OC-T-13	12	alive
ASC-OC-T-7	15	alive	ASC-OC-T-14	12	alive
ASC-OC-T-10	6	dead	ASC-OC-T-15	1	dead

Table S4 – Patient peritoneal metastasis information.

ID	peritoneal metastasis
ASC-GC-B-2	Extensive peritoneal metastasis, M1b: Gastric metastasis
ASC-OC-T-4	omentum majus: 0.9cm, colonic mesentery:0.2cm, splenic hilum:0.5cm, hepatoduodenal ligament:0.3cm
ASC-OC-T-6	Extensive peritoneal metastasis
ASC-OC-T-7	omentum majus: 0.7cm, diaphragm: 3.5cm, hepatoduodenal ligament: 1.5cm, gallbladder peritoneum: 0.3cm, pancreas: 2.5cm, omentum minus:0.2cm, M1b: pleura
ASC-OC-T-10	omentum majus: multiple (3.5cm max), small intestinal plasma membrane: 0.3cm, hepatoduodenal ligament: 0.5cm, hepatic hilar: 1.2cm, small intestinal plasma membrane: 2.5cm
ASC-OC-B-11	Extensive peritoneal metastasis, M1b: pleura
ASC-OC-T-12	omentum majus: 5.5cm, omentum minus: 1.0cm, gallbladder peritoneum: 0.5cm, ileocecal: 3.5cm, bladder peritoneum: 3.5cm, left septum adipose tissue: 9cm, spleen peritoneum: 9.5cm
ASC-OC-T-13	No
ASC-OC-T-14	omental capsule: 0.5cm, colonic mesentery: 0.3cm, splenic peritoneum: 6.5cm, pancreas: 0.3cm
ASC-OC-T-15	omentum majus: 3.2cm, multiple rectal wall: 1.9cm, colonic plasma membrane: 0.5cm, small intestinal plasma membrane: 1cm, colonic plasma membrane: 1.5cm, peripancreatic tissue: 2.8cm, splenic peritoneum: 2.5cm, renal peritoneum: 0.9cm
